# Individual differences in gaze-cuing effect are associated with facial emotion recognition and social conformity

**DOI:** 10.3389/fpsyg.2023.1219488

**Published:** 2023-08-30

**Authors:** Won-Gyo Shin, Hyoju Park, Sung-Phil Kim, Sunhae Sul

**Affiliations:** ^1^Department of Psychology, Pusan National University, Busan, Republic of Korea; ^2^Department of Biomedical Engineering, Ulsan National Institute of Science and Technology, Ulsan, Republic of Korea

**Keywords:** gaze following, gaze-cuing effect, emotion recognition, social conformity, autistic traits, empathy

## Abstract

Spontaneous gaze following and the concomitant joint attention enable us to share representations of the world with others, which forms a foundation of a broad range of social cognitive processes. Although this form of social orienting has long been suggested as a critical starting point for the development of social and communicative behavior, there is limited evidence directly linking it to higher-level social cognitive processes among healthy adults. Here, using a gaze-cuing paradigm, we examined whether individual differences in gaze following tendency predict higher-order social cognition and behavior among healthy adults. We found that individuals who showed greater gaze-cuing effect performed better in recognizing others’ emotion and had greater tendency to conform with group opinion. These findings provide empirical evidence supporting the fundamental role of low-level socio-attentional processes in human sociality.

## Introduction

Humans utilize subtle cues delivered by others, such as gaze, to successfully navigate the complex social world. Gaze allows individuals to align the focus of their attention with others and to form shared representations of the world (Frith and Frith, 2007). Such a process of determining others’ direction of attention and spontaneously following their gaze cues has been suggested to be fundamental not only to the development of infants’ social cognition but also to adults’ socio-cognitive ability (Emery, 2000; Tomasello et al., 2007; Mundy, 2018; Dalmaso et al., 2020). The transient shift of visual attention in following others’ gaze is almost spontaneous and universal, but a considerable range of individual differences has been reported (Bayliss et al., 2005; Hayward and Ristic, 2017). Yet, there is little evidence directly linking the extent to which individuals follow others’ gaze to higher-level social cognitive functions and behaviors, especially among healthy adults. In this exploratory study, we examined whether individual differences in gaze following would be associated with more complex forms of social cognition and behavior in healthy adults.

Following another individual’s gaze direction is closely associated with joint attention, a socio-attentional process in which more than two individuals share attention by looking at the same object (Emery, 2000; Frischen et al., 2007; Yu and Smith, 2013). Overt behavior of sharing attention with others is known to emerge around the first 5–6 months of age (e.g., responsive joint attention by following a caregiver’s pointing or gaze and initiating joint attention by pointing or looking) and is regarded as a critical milestone of social development (Charman, 2004; Mundy and Newell, 2007; Tomasello et al., 2007). Researchers have suggested that the shared visual attention at the early stage of development enables infants to practice perspective taking and mental simulation. These experiences, later, form a basis of the internal representation of others’ minds, i.e., the Theory of Mind (ToM), and the neurocognitive foundations associated with a wide range of complex socio-cognitive functions, such as emotional face processing, empathy, and learning of normative behavior (Mundy, 2018; Stephenson et al., 2021). Longitudinal evidence shows the relationship between gaze following at infancy and ToM at 4.5 years of age (Brooks and Meltzoff, 2015).

In addition, deficits in joint attention processes have been associated with autism spectrum disorder (ASD) (Mundy et al., 1986; Charman, 2004; Dawson et al., 2004). Even among non-clinical populations, variations in eye-gaze patterns have been associated with individual differences in autistic traits (Bayliss et al., 2005; Hayward and Ristic, 2017) and trait empathy (Cowan et al., 2014). Building upon these findings, we expected that self-reported measures of autistic traits and trait empathy would be correlated with variabilities in gaze following patterns, which underlie the individual differences in higher-order social cognition and behavior among healthy adults.

There is indirect evidence that, even in adulthood, gaze following could play a critical role in higher-level socioemotional processes. For instance, accumulated experience of sharing attention is known to facilitate individuals to identify other’s affective states more accurately and sensitively, which in turn enables empathic responses (Mundy, 2018; Stephenson et al., 2021). Supporting this idea, practice of following others’ gaze is reported to significantly improve emotion recognition ability in ASD (Hopkins et al., 2011; Rice et al., 2015). Furthermore, joint attention has been associated with the internalization of social norms and group beliefs (Tomasello and Carpenter, 2007), motivated processes for social engagement and social learning (Mundy and Jarrold, 2010), and behavioral coordination toward common goals and attitudinal consensus (Shteynberg, 2015), which all are closely linked to social conformity. Indeed, children with ASD or typically developing children with greater autistic traits were reported to show decreased tendency of social conformity and impaired social attention was suggested as one of possible factors associated with this finding (Yafai et al., 2014).

In the present study, we aimed to test whether the individual variabilities in socio-attentional processes involving gaze following can be extended to higher-order social cognition and behavior, in particular emotion recognition and social conformity. We leveraged a gaze-cuing paradigm and obtained a quantification of the degree to which each individual’s attention was influenced by others’ gaze while detecting visual targets, i.e., gaze-cuing effect (GCE). First, we tested the association between self-reported measures of social ability (i.e., autistic traits and trait empathy) and the GCE to identify the GCE index that would be most likely to reflect individual differences in social functions. Then, with the specified GCE index, we explored whether individuals with greater tendency to follow others’ gaze would perform better in the emotion recognition task and conform more with group’s opinion in the social conformity task.

## Methods

### Participants

The sample size needed to test our linear multiple regression model was estimated by the power calculations implemented in G*Power (Faul et al., 2007). Assuming a medium effect size of 0.15 and setting a statistical power at 0.85, a sample size of 63 was suggested. Anticipating around 15% drop rate, we recruited 72 healthy undergraduates in the study. Four participants were excluded due to unreliable responses during the experimental tasks (e.g., three participants fell asleep during the task and one participant did not answer at all to the questionnaires), resulting in a sample of 68 participants (30 males, 38 females; mean age 22.09 ± 2.25). Furthermore, prior to our analyses, all data were inspected for outliers exceeding three standard deviations from the sample mean (Howell, 2012). Three outliers, one each from the gaze-cuing task (one female), the emotion recognition task (one male), and the social conformity task (one female), were excluded from the main analysis. These exclusions were made independently of the initial exclusion mentioned earlier and were based on participants’ responses. All the participants gave informed consent in accordance with the protocols approved by the Institutional Review Board and were compensated for their participation.

### Materials and procedure

**Figure 1:**
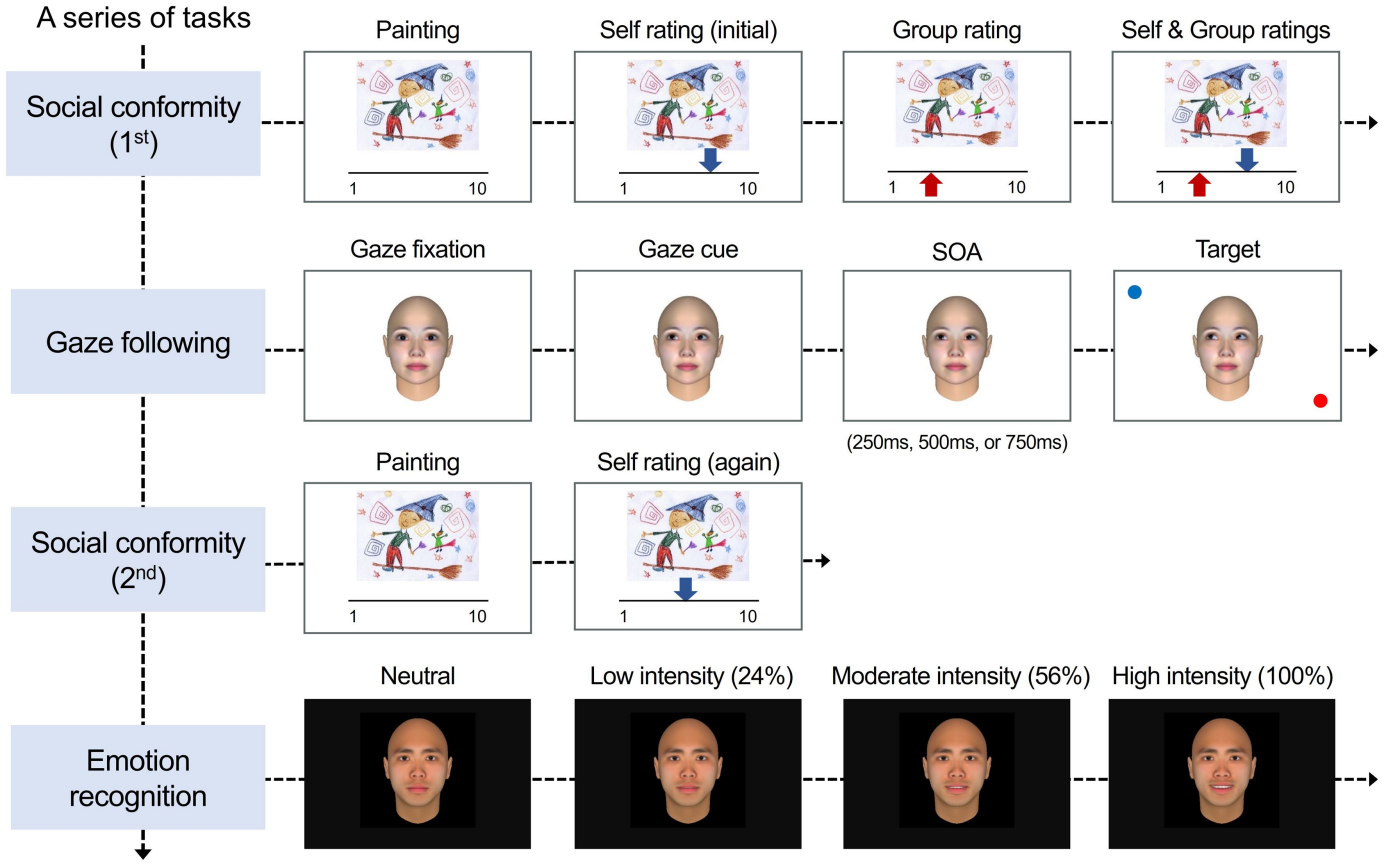
Experimental procedure and stimuli. The experiment consisted of three different tasks in the following sequence: a social conformity task (1st), a gaze-cuing task, the social conformity task (2nd), and an emotion recognition task. In the social conformity task, participants were instructed to rate each painting on a 10-point scale. After rating the painting, the evaluations by self (blue arrow) and by others (i.e., group opinion; red arrow) were presented. In the gaze-cuing task, participants viewed a neutral model with a straight gaze moving their eyes toward left-up, left-down, right-up, or right-down. After different stimulus onset asynchronies (SOA), the target stimulus (red small circle) and a distractor (blue circle) appeared at the same or opposite location of the target’s gaze. Participants were asked to maintain fixation at the center of the screen and to detect the target as exactly and quickly as possible. After completing the gaze-cuing task, participants were unexpectedly asked to rate again the same set of paintings without the presentation of group ratings. In the emotion recognition task, participants were asked to detect and categorize a model’s facial emotions, which were gradually changed from neutral to expressive. Participants were asked to detect the developing emotion as soon as possible when they had the impression to have recognized the target’s emotion and then categorize the recognized emotion. Facial images adapted from FaceGen Modeler software version 3.5: https://facegen.com/.

The experiment consisted of three tasks in the following sequence: the first part of a social conformity task, a gaze-cuing task, the second part of the conformity task (re-evaluation), and an emotion recognition task (Figure 1). All the experimental tasks were programmed and ran on MATLAB (The MathWorks, Natick, MA) using the Psychophysics Toolbox (Version 3 extension; Kleiner et al., 2007).

#### Gaze-cuing task

To measure the gaze-cuing effect, we used a gaze-cuing paradigm (Friesen and Kingstone, 1998). To enable a precise quantification of individuals’ gaze-cuing effects, we manipulated the direction of gaze, the congruency of location between the gaze cue and target, and the interval between the onset of the cue and onset of the target (stimulus onset asynchrony, SOA). We varied the direction of the gaze cue into four peripheral locations in space: left-up, right-up, left-down, and right-down and SOA into relatively short time intervals: 350 ms, 500 ms, and 750 ms.

In the gaze-cuing paradigm, participants detected a target stimulus with a person in the center of the display shifting her gaze. It has been known that even if the direction of the gaze has nothing to do with the target position, participants detect a target faster when it appears in the same position as the gaze direction (congruent condition) than in a position inconsistent with the gaze direction (incongruent condition). The difference in response time between the incongruent condition and the congruent condition in gaze following is known as the GCE because the delayed responses in the incongruent condition can be interpreted as a result of a person’s shifting attention while following another’s gaze (i.e., joint attention with another person).

We used a female model with a neutral facial expression, implemented in the FaceGen 3.5 Modeler (Singular Inversions Inc.). Twenty-six images of the target’s eye movements were generated mimicking a saccadic eye-movement from the front (0%) to the end of gaze direction (100%) to form a movie in which the model made an eye movement toward one of four directions: the left-up, right-up, left-down, or right-down.

Each trial started with a fixation cross in the center of the screen with a duration of 500 ms, followed by the presentation of a female face with a straight gaze for 2000 ms. We included the gaze fixation phase with straight gaze in each trial to facilitate participants’ engagement in the gaze following process and to control for the potential confounding effects of attentional orienting associated with averted gaze stimuli (Adams and Kleck, 2005; Dalmaso et al., 2020). The gaze then gradually shifted to one of the four directions for 310 ms (gaze cue). After either a 250 ms, 500 ms, or 750 ms SOA, a target stimulus (a 1.03 × 1.03 cm red small circle) and a distractor (a 1.03 × 1.03 cm blue circle) appeared 15^∘^ × 8.7^∘^ left-up, right-up, left-down, or right-down with the respect to the center of the screen. The gaze cue, the target, and the distractor remained on the screen until the trial was finished. The target was spatially either congruent (congruent condition) or incongruent (incongruent condition) to the direction of gaze cue with equal probability. The distance between the monitor and the participants was fixed at approximately 57 cm using a headrest.

Participants were instructed to maintain a fixed gaze at the center of the screen throughout the task and to detect the target as exactly and quickly as possible by pressing the corresponding key. The task comprised six within-subject conditions: 2 (type of the previous trial: congruent, incongruent) × 3 (SOA: 250 ms, 500 ms, 750 ms). Each condition consisted of 10 trials, yielding a total of 60 trials. All the trials were randomized across conditions and SOAs. The response times (RT) and accuracy were measured in each trial.

#### Emotion recognition task

Participants were asked to view a face with gradually increasing intensity of facial expression. Their task was to press a spacebar as soon as they detected any emotional expression and categorize the detected emotion. With the gradually changing facial expression, we aimed to measure both the sensitivity and accuracy of emotion recognition. Using the FaceGen 3.5 Modeler (Singular Inversions Inc.), we created two models (one male and one female) showing one of six emotional expressions: pleasure, anger, surprise, disgust, sadness, and fear. To manipulate the intensity of emotional expression, we morphed a neutral (emotional intensity = 0%) face with an expressive face by increments of 4%. As a result, 26 consecutive frames from 0 to 100% intensity were created to form a face whose facial expression increased gradually.

For each trial, a fixation cross was presented for 500 ms in the center of the screen. A neutral face was then presented and then gradually changed to reflect stronger emotional expressions. The first frame on the screen lasted 300 ms and each of the subsequent 25 frames also occupied the screen for 300 ms. Participants were asked to detect the developing emotion as soon as possible and to press the space bar when they felt they recognized any emotion. Once participants had pressed the space bar, the facial expression stopped changing. Subsequently, the participants were required to choose one of the six emotion categories that best described the detected emotion by pressing the corresponding key. Faces were presented in random order and remained on the screen until the participants had made their responses. A total of 60 trials were included: 2 models (female and male) × 6 emotions (pleasure, anger, surprise, disgust, sadness, and fear) × 5 trials. The accuracy and RTs (sensitivity) were measured in each trial.

#### Social conformity task

The social conformity task consisted of two parts: evaluation and re-evaluation. Participants were asked to evaluate children’s paintings, and then the mean ratings of the other participants were presented. For a stimulus set to be evaluated, we created 50 paintings that mimicked paintings of elementary school students. Based on 10 independent raters’ evaluation, 20 paintings with the highest variances in rating scores were selected and included in the social conformity task. The purpose of selecting the paintings with higher rating variance was to avoid participants’ doubt when group ratings were inconsistent with their own ratings.

In the evaluation phase, participants were instructed to imagine they were a judge of a drawing contest for elementary school students and to evaluate the paintings on a 10-point scale, ranging from poor (1) to excellent (10). In each trial, participants evaluated a painting by moving an arrow (blue) on a rating slider with the left and right arrow keys on the keyboard. After 2000 ms, a mean rating of the other participants (red arrow) appeared below the scale for 3,000 ms. The mean ratings of the other participants (i.e., group opinion) were, in fact, generated by a computer algorithm. Group opinion matched the participant’s rating in 40% of the trials. In the remaining 60% of trials, group opinion differed from participant’s rating. The gap between the participants’ rating and group opinion was pseudorandomly varied by ±1, ±2, or ± 3 points, using an adaptive algorithm that kept the overall ratio of more negative or more positive group ratings approximately equal across the trials (Klucharev et al., 2009). Both the individual participants’ and the group rating remained on the screen for an additional 3,000 ms.

After completing the gaze-cuing task, participants were unexpectedly asked to rate the same set of paintings again without the presentation of group ratings. The order of stimuli was randomized across subjects. The rating scores from the first phase and the second phase given by each participant were measured to identify the extent to which participants adjusted their opinion in accordance with the group’s opinion (i.e., conformity effect).

#### Self-reported measures of autistic traits and empathy

Participants’ autistic traits were measured using the Autism Quotient (AQ; Baron-Cohen et al., 2001) that comprises five subscales: social skill, communication, imagination, attention to detail, and attention-switching. The total score of the AQ subscales is known to reflect autistic traits in the normal population with a higher score indicating a stronger disposition toward autism (Baron-Cohen et al., 2001). To assess trait empathy, we used the Empathy Quotient (EQ; Lawrence et al., 2004), which measures the cognitive, affective, and behavioral aspects of empathy, with a higher score reflecting greater dispositional empathy. To control for the effects of anxiety and depression on our dependent variables, we included the CES-D (Radloff, 1977) and GAD-7 (Spitzer et al., 2006).

### Data analyses

#### Gaze-cuing effect

The raw RTs of each participant were then transformed into *z*-score to minimize the effect of individual variabilities on the GCE. To measure individual differences in the GCE, we calculated a GCE score for each participant by subtracting the mean RT on congruent trials from the mean RT on incongruent trials. In the gaze-cuing paradigm, it is typically expected that the RTs in the congruent trials should be shorter than the RTs in the incongruent trials because the gaze of a target person guides participants’ attention. Therefore, greater GCE scores indicate greater sensitivity to social gaze. Also, previous studies have suggested that subtle difference in spontaneous gaze following can be affected by the condition of previous trials as well as SOA (Qian et al., 2020; Narganes-Pineda et al., 2022). To capture these nuanced variations, we calculated GCE scores for the condition of previous trials (congruent or incongruent) and SOA (250 ms, 500 ms, or 750 ms), separately, yielding a total of six GCE scores for each participant. This approach provides a more refined analysis of examining GCE under different experimental conditions.

#### Emotion recognition performance

Two measures of emotion recognition performance were derived: emotion recognition accuracy and emotion recognition sensitivity. First, the accuracy score was calculated based on signal detection theory (SDT; Green and Swets, 1966). To assess the ability to distinguish one specific emotion from other emotions, we used hit-rates (HR) and false alarm rates (FAR). HRs and FARs were adjusted to a range between 0.0001 and 0.9999 to avoid problems resulting from infinite values. The discriminability index (*d*’) was calculated using the following (Macmillan and Creelman, 1990):


$$d^{’}=z\;\left(HR\right)–z\;\left(FAR\right).$$

Higher values of *d*’ indicate better ability to differentiate the target emotion from non-target emotions, namely greater accuracy. We used the overall accuracy score irrespective of emotion categories as a main dependent variable in the analyses but also reported the results for each emotion category in the online Supplementary material.

Second, emotion recognition sensitivity was measured using RTs of the HR responses, with fast responses indicating greater sensitivity in accurately detecting the target emotion with lower intensity of expression. Log-transformed RTs were used in the analyses. Like the accuracy score, overall RTs across emotion categories were used as the main dependent variable but the results for each emotion category were included in the online Supplementary material.

#### Social conformity effect

To quantify each individual’s susceptibility to the group norm, we first calculated (i) difference scores between participant’s first rating scores and the group’s rating scores (opinion discrepancy) and (ii) difference scores between participants’ second rating scores and their first rating scores (opinion change) for each participant. Then, we derived a standardized regression coefficient (Beta) from the linear regression model predicting opinion change from opinion discrepancy. The Beta of the opinion discrepancy reflects the extent to which an individual adjusts their evaluation to the group’s opinion, and a higher value indicates a greater propensity to change one’s opinion to match the group mean. We operationalized the conformity effect with the Beta coefficients. All the descriptive statistics including the GCE, emotion recognition performance, and social conformity effect are presented in Supplementary Tables S1, S2.

#### Statistical analyses

First, we confirmed the general effect of gaze-cuing using one-sample *t*-tests on the six GCE scores. Second, we performed a 2 (type of the previous trial: congruent vs. incongruent) × 3 (SOA: 250 ms, 500 ms, 750 ms) repeated measures analysis of variance (ANOVA) on the GCE scores to examine the overall effect of previous trials and SOA. Third, we identified a GCE index that reflected individual differences in both autistic traits and trait empathy based on multiple regression analyses, Pearson’s correlations, and multivariate multiple regression analysis.

Lastly, with the identified GCE index, we performed the multiple regression analyses to test our hypotheses that individual differences in the GCE would predict emotion recognition performance and susceptibility to group opinion. The effects of age, gender, depression, and anxiety were controlled for all the regression analyses. Data were analyzed using R statistical software (Version 3.3.3; The R foundation) and SPSS Statistics (Version 25; IBM Corporation). All statistical tests were two-sided and *p* < 0.05 was considered significant.

## Results

### Gaze-cuing effect

We examined the general effects of experimental conditions on GCE using a 2 (type of the previous trial: congruent vs. incongruent) × 3 (SOA: 250 ms, 500 ms, 750 ms) repeated measures analysis of variance (ANOVA). There was a significant main effect of the type of previous trial [*F*_(1, 65)_ = 6.535, *p* = 0.013] and the GCE was greater than zero only when the gaze cue in the previous trial was congruent with the target position (see Supplementary Table S1 for the descriptive statistics). The main effect of SOA [*F*_(2,64)_ = 0.310, *p* = 0.735] and the two-way interaction effect [*F*_(2, 64)_ = 0.104, *p* = 0.901] were not significant. Additional one-sample *t*-tests showed variations of the GCE across different SOAs when the gaze cue in the previous trial was congruent. Specifically, the GCEs at the SOA of 250 ms and 750 ms were significant (*p* = 0.010 and *p* = 0.011, respectively), while the GCE at the SOA of 500 ms was not statistically significant (*p* = 0.095; Supplementary Table S1).

### AQ and EQ predicted GCE

**Figure 2:**
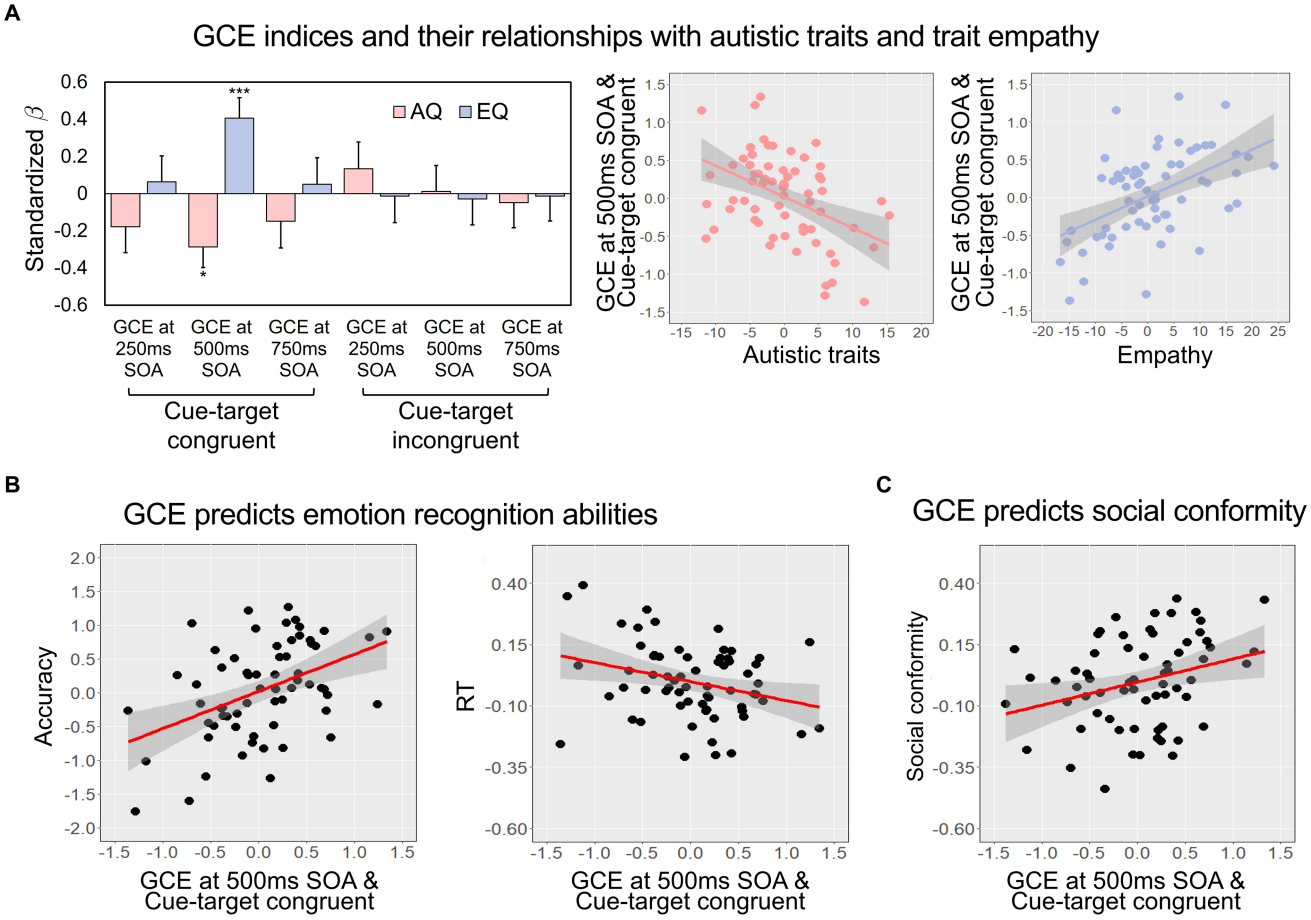
**(A)** The regression coefficients of AQ and EQ predicting GCE indices, separately presented according to the condition of previous trials (congruent or incongruent) and SOA (250 ms, 500 ms, or 750 ms) (left), and the partial regression plots showing the significant relationships of the GCE index measured when the SOA was 500 ms and the cue and target were consistent in the previous trial with the AQ and EQ (right). Error bars represent standard errors of the mean. **(B,C)** The partial regression plots showing the relationship of the GCE with emotion recognition and social conformity. The GCE index measured when the SOA was 500 ms and the cue and target were consistent in the previous trial significantly predicted both the accuracy and sensitivity (shorter RT means higher sensitivity) in the emotion recognition task **(B)** and social conformity **(C)**. The gray shadings indicate 95% confidence intervals. AQ, autism quotient; EQ, empathy quotient; GCE, gaze-cuing effect; RT, response time; SOA, stimulus onset asynchrony. ***p < 0.001, *p < 0.05.

To examine the association between the gaze-cuing effect and individual differences in autistic traits and trait empathy, we performed multiple regression analyses with the GCE of each condition as an outcome variable and the AQ and EQ scores as predictors. Among the six GCE indices, we found the GCE measured when the type of the previous trial was congruent and SOA was 500 ms had significant relationships of AQ (*β* = −0.287, *t* = −2.581, *p* = 0.012) and EQ (*β* = 0.406, *t* = 3.660, *p* < 0.001; Figure 2A and Supplementary Table S3-1; For details of Pearson’s correlations, see Supplementary Table S3-2). These results remained significant even after accounting for age, gender, depression, and anxiety. Multivariate multiple regression analysis also showed significant relationships of AQ and EQ with the GCE measured when the type of the previous trial was congruent and SOA was 500 ms (for details, see Supplementary Table S3-3). Based on this result, we expected that the GCE measured when the type of the previous trial was congruent and SOA was 500 ms would be most likely to be associated with emotion recognition performance and social conformity.

### GCE predicts emotion recognition performance

We performed a multiple regression analysis with the GCE index predicting overall emotion recognition accuracy (i.e., the averaged *d*’ value). Consistent with the results with the AQ and EQ, we found that the GCE measured when the type of the previous trial was congruent and SOA was 500 ms significantly predicted the accuracy of emotion recognition [*R*^2^ = 0.291, Adjusted *R*^2^ = 0.231, *F*_(5, 60)_ = 4.915, *p* < 0.001, *β* = 0.508, *t* = 4.361, *p* < 0.001; Figure 2B left]. Moreover, a multiple regression model predicting overall RT [*R*^2^ = 0.209, Adjusted *R*^2^ = 0.143, *F*_(5, 60)_ = 3.162, *p* = 0.013] revealed that participants with greater GCE in the same condition showed greater sensitivity (i.e., lower RT; *β* = −0.325, *t* = −2.638, *p* = 0.011, Figure 2B right). In both regression models, age, depression, and anxiety did not have a significant relationship with the GCE index, while the influence of gender on overall RT was observed (Supplementary Tables S4, S5). The results of the separate analyses on different emotions are reported in Supplementary Tables S4, S5 and these results are discussed in the online Supplementary material. None of the GCE indices measured when SOA was 250 ms or 750 ms were predictive of either the accuracy or the RTs (Supplementary Tables S6).

### GCE predicts social conformity

Lastly, we examined whether the GCE predicted individual differences in social conformity. Consistent with the results described above, the multiple regression model predicting the conformity with the GCE measured when the type of the previous trial was congruent and SOA was 500 ms [*R*^2^ = 0.212, Adjusted *R*^2^ = 0.147, *F*_(5, 60)_ = 3.236, *p* = 0.012; Figure 2C] showed that participants with greater GCE were more likely to adjust their judgment according to group’s opinion (*β* = 0.266, *t* = 2.160, *p* = 0.035). No other factors, including age, gender, depression, and anxiety, significantly predicted the conforming tendency (Supplementary Table S7). The GCE indices measured when SOA was 250 ms or 750 ms were not associated with the tendency of conformity (Supplementary Table S8).

## Discussion

Though gaze following has long been hypothesized to lay the foundation of human sociality, little research has directly examined the link between the gaze following and higher-order social cognition and behavior in healthy adults. By exploring this relationship using the well-established gaze-cuing paradigm, the present study shows that individual differences in gaze following are associated with the ability to recognize others’ emotions and tendency to conform with group norm.

First, we found that GCE measured when the SOA was 500 ms and the cue was reliable in the previous trial was consistently associated with both self-reported measures of autistic trait and trait empathy, and behavioral measures of emotion recognition and social conformity. These findings are in line with prior research highlighting the relationships of individual variabilities in eye-gaze patterns with autistic traits (Bayliss et al., 2005; Hayward and Ristic, 2017) and trait empathy (Cowan et al., 2014).

In the emotion recognition task, participants with greater GCE showed higher accuracy and sensitivity, supporting the facilitating role of shared attention in processing social information (Mundy and Newell, 2007). Individuals with greater sensitivity to other’s gaze may be more likely to pay attention to others’ faces conveying rich social information, which would possibly allow them to better infer others’ emotional states from their facial expressions. From a developmental perspective, more frequent experiences of joint attention at early developmental stages may be associated with more frequent experiences of processing facial emotions, which in turn may result in individual differences in both social attention and emotion recognition. Future research probing common and distinct neurodevelopmental underpinnings of joint attention and facial emotion recognition (see Pitcher and Ungerleider, 2021) and tracking eye movements during the tasks could test these possibilities.

We also found that participants with greater GCE were more likely to conform with group opinion. Considering that joint attention has been associated with social learning (Tomasello and Carpenter, 2007; Mundy and Jarrold, 2010) and that our brain’s reward system is involved in both social conformity and joint attention (Oberwelland et al., 2016; Olsson et al., 2020), the rewarding quality of social engagement via joint attention and conformity might underly this relationship. Yet, it is unclear whether GCE reflects general tendency to pay attention to social information including others’ opinions or individuals who are more likely to align their attention with others have greater tendency to align their behavior with others. Where the difference lies could be addressed in future research by measuring both perception of norm and behavioral adjustment.

It is noteworthy that only the GCE derived from the trials where the SOA was 500 ms and the cue and target were congruent in the previous trial was predictive of the social cognition measures (i.e., EQ, AQ, emotion recognition, and social conformity). This finding is in line with previous studies reporting that the gaze-cuing effect depended on the cue-target congruency (Kompatsiari et al., 2022) and that the gaze-cuing effect at the similar range of SOA (300 ~ 700 ms) was related to personal dispositions (Carraro et al., 2015) or contextual effects (McCrackin and Itier, 2019). Though further investigations into the specific conditions that best reflect individual differences in social cognition and behavior are needed, our findings suggest a possibility of adopting the gaze-cuing paradigm as a useful non-verbal tool for measuring basic social ability.

Interestingly, when we examined the average GCEs when the cue was reliable in the previous trial, the average GCE at the SOA of 500 ms was not significantly greater than zero, unlike the significant GCEs observed at the SOAs of 250 ms or 750 ms. We suspect that this might be due to relatively greater individual variabilities in the GCEs at the SOA of 500 ms, which might be associated with greater sensitivity to capture individual differences in social behaviors. Indeed, only the GCEs at the SOA of 500 ms consistently showed significant relationships with all our self-reported and behavioral measures. That is, the GCEs at the SOA of 500 ms seem to tap into the individual differences in the specific aspects of social functions that could be measured by the self-reported AQ and EQ, and the behavioral measures of emotion recognition and social conformity used in the present study. There is also a possibility that the GCEs at different time windows reflect different social functions. Considering the gap between typical gaze-cuing experiments and the gaze-cuing effect involving real-life social contexts as highlighted in the literature (Frischen and Tipper, 2004), exploring how temporal dynamics of the socio-attentional processes interact with different social functions will provide a more comprehensive understanding of human social behavior from basic to higher-order processes.

Although we controlled for the effects of gender in the main analyses, it would be worth mentioning the significant gender effects observed in the GCE and emotion recognition, considering previous research reporting gender effects in gaze following (Bayliss et al., 2005), emotion recognition (Thompson and Voyer, 2014), and social conformity (Cross et al., 2017). In the present study, women exhibited shorter overall RTs than men when accurately detecting the target emotions (Supplementary Table S5). This aligns with previous research that women exhibited better performance than men in the tasks involving the ability to recognize non-verbal displays of emotions (Thompson and Voyer, 2014). We also observed greater GCE among men than women (Supplementary Tables S3-1, S3-3), which contrasts with prior work indicating greater GCE among women (Bayliss et al., 2005). This inconsistency may arise from various factors including methodological variations and cultural norms. Accounting for the factors related to gender effects would foster a more comprehensive understanding of the observed findings.

Lastly, caution is needed when interpreting our results because they are correlational in nature. There is a possibility that the associations between the GCE, self-reported AQ and EQ, and behaviorally measured higher-order social cognition could be commonly resulted from a third factor. For instance, recent evidence suggested that individual differences in gaze following can be significantly influenced by the broad autism phenotype (BAP) in healthy adults (Swanson and Siller, 2014; Whyte and Scherf, 2018). Future studies may benefit from considering this possibility and offer a more precise understanding of the causal relationships among the GCE and different levels of social behavior.

In sum, the present study shows that gaze following may continuously exert influence on diverse human social functions even after early developmental stage. By carefully manipulating the gaze-cuing paradigm and establishing associations between GCE indices and basic social abilities, we have identified a behavioral marker that sensitively captures individual differences in high-order social cognition and behavior. These findings contribute to the experimental psychology literature by providing empirical evidence that emphasizes the fundamental role of socio-attentional processes in human sociality. Additionally, our study has potential implications for informing the development of interventions for enhancing social cognitive abilities in clinical populations, such as individuals with ASD, as well as the early detection of individuals with subclinical autistic traits.

## Data availability statement

The study materials, data, code for all analyses are available at http://osf.io/w968f/. The full dataset is not publicly available due to lack of informed consent and ethical approval but is available on request by qualified scientists.

## Ethics statement

All the participants gave informed consent in accordance with the protocols approved by the Institutional Review Board at Pusan National University and were compensated for their participation.

## Author contributions

S-PK and SS designed the study. HP collected the data. W-GS and HP analyzed the data. W-GS and SS wrote the manuscript. HP and S-PK provided helpful comments about the results and all authors approved the final version of the manuscript.

## Funding

This work was supported by the Korea Health Technology R&D Project through the Korea Health Industry Development Institute (KHIDI) funded by the Ministry of Health & Welfare (No. HI18C1180 to SS) and the National Research Foundation of Korea (NRF) grant funded by the Korea government (MSIT) (No. NRF-2020M3E5D908078721 to S-PK and SS and 2021R1C1C2008426 to W-GS).

## Conflict of interest

The authors declare that the research was conducted in the absence of any commercial or financial relationships that could be construed as a potential conflict of interest.

## Publisher’s note

All claims expressed in this article are solely those of the authors and do not necessarily represent those of their affiliated organizations, or those of the publisher, the editors and the reviewers. Any product that may be evaluated in this article, or claim that may be made by its manufacturer, is not guaranteed or endorsed by the publisher.
