# Supplementary material for: Individual differences in gaze-cuing effect are associated with facial emotion recognition and social conformity
Source: Front Psychol. 2023 Aug 30;14:1219488. doi: 10.3389/fpsyg.2023.1219488 (PMC10499521; doi:10.3389/fpsyg.2023.1219488)
Supplement: Supplementary file 1 [file Data_Sheet_1.docx]

**Supplementary material**

**Individual differences in gaze-cuing effect are associated with facial emotion recognition and social conformity**

Won-Gyo Shin^1^, Hyoju Park^1^, Sung-Phil Kim^2^ and Sunhae Sul^1^*

^1^ Department of Psychology,
Pusan National University, Busan,
Republic of Korea

^2^ Department of Biomedical Engineering, Ulsan,

UNIST,

Republic of Korea

*Corresponding author:
ssul@pusan.ac.kr
Social Neuroscience Laboratory
Department of Psychology
Pusan National University,
2 Busandaehakro-63beon-gil,
Geumjeong-gu, Busan, 46241, Republic of Korea
+82-51-510-2191

**Supplementary Table 1**. Mean and *SD* of the six GCE indices.

| **Cue-target congruency in the Previous trial** | **SOA** | **Mean (*SD*)** | **One-sample T-test** | | |
| --- | --- | --- | --- | --- | --- |
|  |  |  | ***t* _(_*_df_*_)_** | ***P*** | ***Cohen’s d*** |
| Congruent | 250ms | .204 (.632) | 2.644 _(_*_66_*_)_ | .010 | .323 |
| Congruent  c | 500ms | .134 (.650) | 1.693 _(_*_66_*_)_ | .095 | .207 |
| Congruent | 750ms | .199 (.622) | 2.612 _(_*_66_*_)_ | .011 | .319 |
| Incongruent | 250ms | .033 (.706) | .384 _(_*_66_*_)_ | .702 | .047 |
| Incongruent | 500ms | -.004 (.689) | -.044 _(_*_66_*_)_ | .965 | -.005 |
| Incongruent | 750ms | .003 (.571) | .036 _(_*_65_*_)_ | .971 | .004 |

*Note*. GCE, Gaze-cuing effect; SOA, stimulus onset asynchrony.

**Supplementary Table 2**. Mean and *SD* of the measures from the emotion recognition task and social conformity task.

|  | **Measures** | **Mean** | ***SD*** |
| --- | --- | --- | --- |
| **Emotion recognition task** | | |  |
| Accuracy | Overall | 2.723 | 0.766 |
|  | Anger | 3.533 | 1.421 |
|  | Disgust | 1.704 | 1.494 |
|  | Sadness | 1.885 | 1.065 |
|  | Fear | 1.482 | 1.547 |
|  | Surprise | 1.837 | 0.856 |
|  | Pleasure | 5.863 | 1.526 |
| Hit rate | Overall | 0.617 | 0.104 |
|  | Anger | 0.897 | 0.121 |
|  | Disgust | 0.497 | 0.282 |
|  | Sadness | 0.465 | 0.197 |
|  | Fear | 0.281 | 0.187 |
|  | Surprise | 0.614 | 0.208 |
|  | Pleasure | 0.945 | 0.121 |
| False alarm rate | Overall | 0.057 | 0.019 |
|  | Anger | 0.028 | 0.030 |
|  | Disgust | 0.051 | 0.041 |
|  | Sadness | 0.015 | 0.020 |
|  | Fear | 0.087 | 0.044 |
|  | Surprise | 0.106 | 0.067 |
|  | Pleasure | 0.059 | 0.045 |
| Response time | Overall | 1.551 | 0.173 |
|  | Anger | 1.428 | 0.220 |
|  | Disgust | 1.639 | 0.209 |
|  | Sadness | 1.716 | 0.176 |
|  | Fear | 1.694 | 0.194 |
|  | Surprise | 1.622 | 0.200 |
|  | Pleasure | 1.411 | 0.239 |
| **Social conformity task** | | | |
| Social conformity |  | 0.216 | 0.197 |

**Supplementary Table 3-1**. Regression results of the six GCE indices as outcome variables, AQ and EQ as predictors, and age, gender, depression, and anxiety as covariates.

| **Dependent**  **Variables** | **Independent**  **Variables** | **B** | ***SE*** | ***β*** | ***t*** | ***P*** |
| --- | --- | --- | --- | --- | --- | --- |
| **Cue-target congruent in the previous trial** | | | | | | |
| GCE at 250ms SOA | (Constant) | -.083 | .938 |  | -.088 | .930 |
|  | AQ | -.017 | .014 | -.178 | -1.268 | .210 |
|  | EQ | .004 | .009 | .064 | .455 | .651 |
|  | Age | .029 | .037 | .103 | .793 | .431 |
|  | Gender | -.062 | .169 | -.049 | -.368 | .714 |
|  | Depression | -.012 | .011 | -.226 | -1.169 | .247 |
|  | Anxiety | .027 | .032 | .159 | .851 | .398 |
| GCE at 500ms SOA | (Constant) | <.001 | .765 |  | <.001 | 1.000 |
|  | AQ | -.029 | .011 | -.287 | -2.581 | .012 |
|  | EQ | .025 | .007 | .406 | 3.660 | <.001 |
|  | Age | .004 | .030 | .013 | .129 | .898 |
|  | Gender | -.314 | .138 | -.242 | -2.281 | .026 |
|  | Depression | -.003 | .009 | -.060 | -.392 | .696 |
|  | Anxiety | .016 | .026 | .090 | .609 | .545 |
| GCE at 750ms SOA | (Constant) | .345 | .952 |  | .362 | .719 |
|  | AQ | -.014 | .014 | -.148 | -1.023 | .310 |
|  | EQ | .003 | .009 | .050 | .348 | .729 |
|  | Age | .009 | .037 | .033 | .246 | .807 |
|  | Gender | -.127 | .171 | -.102 | -.742 | .461 |
|  | Depression | .001 | .011 | .025 | .124 | .902 |
|  | Anxiety | -.007 | .032 | -.040 | -.206 | .838 |
| **Cue-target incongruent in the previous trial** | | | | | | |
| GCE at 250ms SOA | (Constant) | 1.338 | 1.079 |  | 1.240 | .220 |
|  | AQ | .014 | .016 | .134 | .922 | .360 |
|  | EQ | -.001 | .010 | -.013 | -.091 | .927 |
|  | Age | -.052 | .042 | -.165 | -1.231 | .223 |
|  | Gender | -.175 | .194 | -.124 | -.901 | .371 |
|  | Depression | -.007 | .012 | -.122 | -.613 | .542 |
|  | Anxiety | <.001 | .036 | .002 | .010 | .992 |
| GCE at 500ms SOA | (Constant) | .175 | 1.020 |  | .172 | .864 |
|  | AQ | .001 | .015 | .012 | .088 | .930 |
|  | EQ | -.002 | .009 | -.029 | -.206 | .837 |
|  | Age | .018 | .040 | .059 | .457 | .649 |
|  | Gender | -.347 | .184 | -.252 | -1.887 | .064 |
|  | Depression | -.014 | .011 | -.239 | -1.235 | .222 |
|  | Anxiety | .058 | .034 | .317 | 1.694 | .095 |
| GCE at 750ms SOA | (Constant) | .542 | .817 |  | .663 | .510 |
|  | AQ | -.004 | .012 | -.048 | -.353 | .725 |
|  | EQ | -.001 | .007 | -.013 | -.096 | .924 |
|  | Age | -.011 | .032 | -.043 | -.338 | .736 |
|  | Gender | -.260 | .149 | -.228 | -1.739 | .087 |
|  | Depression | -.006 | .009 | -.130 | -.692 | .492 |
|  | Anxiety | .076 | .027 | .498 | 2.748 | .008 |

*Note*. AQ, Autism-spectrum quotient; EQ, Empathy quotient; GCE, Gaze-cuing effect; SOA, stimulus onset asynchrony.

**Supplementary Table 3-2**. The correlation results of the relationship of the six GCE indices with AQ and EQ.

| **Cue-target congruency in the Previous trial** | **SOA** | **AQ** | | **EQ** | |
| --- | --- | --- | --- | --- | --- |
|  |  | ***r*** | ***P*** | ***r*** | ***P*** |
| Congruent | 250ms | -0.232 | 0.059 | 0.194 | 0.116 |
| Congruent  c | 500ms | -0.489 | < 0.001 | 0.537 | < 0.001 |
| Congruent | 750ms | -0.185 | 0.134 | 0.110 | 0.376 |
| Incongruent | 250ms | 0.065 | 0.600 | -0.055 | 0.660 |
| Incongruent | 500ms | -0.021 | 0.866 | 0.025 | 0.838 |
| Incongruent | 750ms | -0.034 | 0.787 | 0.027 | 0.831 |

*Note*. AQ, Autism-spectrum quotient; EQ, Empathy quotient; GCE, Gaze-cuing effect; SOA, stimulus onset asynchrony.

**Supplementary Table 3-3**. Multivariate multiple regression results of the six GCE indices as outcome variables, AQ and EQ as predictors, and age, gender, depression, and anxiety as covariates.

| **Dependent**  **Variables** | **Independent**  **Variables** | **B** | ***SE*** | ***t*** | ***P*** |
| --- | --- | --- | --- | --- | --- |
| **Cue-target congruent in the previous trial** | | | | | |
| GCE at 250ms SOA | (Constant) | -.024 | .942 | -.026 | .979 |
|  | AQ | -.016 | .014 | -1.168 | .247 |
|  | EQ | .004 | .009 | .470 | .640 |
|  | Age | .026 | .037 | .704 | .485 |
|  | Gender | -.092 | .172 | -.532 | .597 |
|  | Depression | -.011 | .011 | -1.034 | .305 |
|  | Anxiety | .026 | .032 | .817 | .417 |
| GCE at 500ms SOA | (Constant) | .051 | .767 | .066 | .948 |
|  | AQ | -.027 | .011 | -2.468 | .016 |
|  | EQ | .026 | .007 | 3.676 | .001 |
|  | Age | .001 | .030 | .037 | .970 |
|  | Gender | -.340 | .140 | -2.425 | .018 |
|  | Depression | -.002 | .009 | -.256 | .799 |
|  | Anxiety | .015 | .026 | .574 | .568 |
| GCE at 750ms SOA | (Constant) | .349 | .962 | .362 | .718 |
|  | AQ | -.014 | .014 | -1.004 | .320 |
|  | EQ | .003 | .009 | .347 | .730 |
|  | Age | .009 | .038 | .237 | .814 |
|  | Gender | -.129 | .176 | -.735 | .465 |
|  | Depression | .001 | .011 | .130 | .897 |
|  | Anxiety | -.007 | .032 | -.206 | .837 |
| **Cue-target incongruent in the previous trial** | | | | | |
| GCE at 250ms SOA | (Constant) | 1.357 | 1.090 | 1.245 | .218 |
|  | AQ | .015 | .016 | .937 | .353 |
|  | EQ | -.001 | .010 | -.086 | .931 |
|  | Age | -.053 | .043 | -1.240 | .220 |
|  | Gender | -.185 | .199 | -.927 | .358 |
|  | Depression | -.007 | .012 | -.568 | .572 |
|  | Anxiety | <.001 | .037 | .001 | .999 |
| GCE at 500ms SOA | (Constant) | .194 | 1.030 | .188 | .852 |
|  | AQ | .002 | .015 | .113 | .910 |
|  | EQ | -.002 | .009 | -.200 | .842 |
|  | Age | .017 | .040 | .428 | .670 |
|  | Gender | -.356 | .188 | -1.889 | .064 |
|  | Depression | -.014 | .012 | -1.179 | .243 |
|  | Anxiety | .058 | .035 | 1.671 | .100 |
| GCE at 750ms SOA | (Constant) | .542 | .817 | .663 | .510 |
|  | AQ | -.004 | .012 | -.353 | .725 |
|  | EQ | -.001 | .007 | -.096 | .924 |
|  | Age | -.011 | .032 | -.338 | .736 |
|  | Gender | -.260 | .149 | -1.739 | .087 |
|  | Depression | -.006 | .009 | -.692 | .492 |
|  | Anxiety | .076 | .027 | 2.748 | .008 |

*Note*. AQ, Autism-spectrum quotient; EQ, Empathy quotient; GCE, Gaze-cuing effect; SOA, stimulus onset asynchrony.

**Supplementary Table 4.** Regression results of the accuracy scores of emotion recognition as outcome variables and the GCE at the SOA of 500ms when the gaze cue in the previous trial was congruent with the target position as a predictor, and age, gender, depression, and anxiety, as covariates.

| **Dependent**  **Variables** | **Independent**  **Variables** | **B** | ***SE*** | ***β*** | ***t*** | ***P*** |
| --- | --- | --- | --- | --- | --- | --- |
| Overall accuracy | (Constant) | 2.251 | .997 |  | 2.258 | .028 |
|  | GCE | .604 | .138 | .508 | 4.361 | <.001 |
|  | Age | .002 | .040 | .006 | .052 | .959 |
|  | Gender | .344 | .185 | .223 | 1.858 | .068 |
|  | Depression | .005 | .011 | .080 | .477 | .635 |
|  | Anxiety | -.065 | .034 | -.316 | -1.922 | .059 |
| Anger | (Constant) | 3.073 | 2.123 |  | 1.448 | .153 |
|  | GCE | .280 | .295 | .128 | .949 | .347 |
|  | Age | .019 | .086 | .029 | .223 | .824 |
|  | Gender | .076 | .394 | .027 | .193 | .847 |
|  | Depression | .022 | .024 | .176 | .911 | .366 |
|  | Anxiety | -.117 | .072 | -.307 | -1.619 | .111 |
| Disgust | (Constant) | 2.510 | 2.215 |  | 1.133 | .262 |
|  | GCE | .474 | .308 | .205 | 1.542 | .128 |
|  | Age | -.020 | .090 | -.029 | -.224 | .823 |
|  | Gender | -.297 | .411 | -.099 | -.723 | .472 |
|  | Depression | .026 | .025 | .203 | 1.064 | .292 |
|  | Anxiety | -.090 | .075 | -.226 | -1.203 | .234 |
| Sadness | (Constant) | 3.038 | 1.524 |  | 1.993 | .051 |
|  | GCE | .453 | .212 | .275 | 2.137 | .037 |
|  | Age | -.074 | .062 | -.147 | -1.199 | .235 |
|  | Gender | .431 | .282 | .201 | 1.530 | .131 |
|  | Depression | -.023 | .017 | -.252 | -1.343 | .184 |
|  | Anxiety | .035 | .052 | .124 | .677 | .501 |
| Fear | (Constant) | -1.569 | 2.140 |  | -.733 | .466 |
|  | GCE | .734 | .297 | .307 | 2.467 | .016 |
|  | Age | .077 | .087 | .106 | .887 | .378 |
|  | Gender | 1.091 | .397 | .351 | 2.745 | .008 |
|  | Depression | -.033 | .024 | -.247 | -1.388 | .170 |
|  | Anxiety | .024 | .073 | .059 | .336 | .738 |
| Surprise | (Constant) | 1.425 | 1.220 |  | 1.168 | .248 |
|  | GCE | .499 | .164 | .383 | 3.038 | .004 |
|  | Age | -.015 | .049 | -.038 | -.312 | .756 |
|  | Gender | .539 | .225 | .313 | 2.392 | .020 |
|  | Depression | -.012 | .013 | -.159 | -.882 | .381 |
|  | Anxiety | .009 | .041 | .038 | .212 | .833 |
| Pleasure | (Constant) | 4.789 | 2.049 |  | 2.338 | .023 |
|  | GCE | 1.052 | .285 | .444 | 3.695 | <.001 |
|  | Age | .030 | .083 | .042 | .362 | .719 |
|  | Gender | .312 | .380 | .102 | .821 | .415 |
|  | Depression | .034 | .023 | .255 | 1.481 | .144 |
|  | Anxiety | -.181 | .070 | -.442 | -2.603 | .012 |

*Note*. GCE, Gaze-cuing effect; SOA, stimulus onset asynchrony.

**Supplementary Table 5.** Regression results of the RTs of emotion recognition (sensitivity) as outcome variables and the GCE at the SOA of 500ms when the gaze cue in the previous trial was congruent with the target position as a predictor, and age, gender, depression, and anxiety as covariates.

| **Dependent**  **Variables** | **Independent**  **Variables** | **B** | ***SE*** | ***β*** | ***t*** | ***P*** |
| --- | --- | --- | --- | --- | --- | --- |
| Overall RT | (Constant) | 1.649 | .222 |  | 7.429 | <.001 |
|  | GCE | -.081 | .031 | -.325 | -2.638 | .011 |
|  | Age | .006 | .009 | .079 | .672 | .504 |
|  | Gender | -.129 | .041 | -.396 | -3.127 | .003 |
|  | Depression | -.001 | .002 | -.100 | -.564 | .575 |
|  | Anxiety | .003 | .008 | .058 | .334 | .739 |
| Anger | (Constant) | 1.830 | .288 |  | 6.358 | <.001 |
|  | GCE | -.113 | .040 | -.348 | -2.830 | .006 |
|  | Age | -.004 | .012 | -.037 | -.317 | .752 |
|  | Gender | -.172 | .053 | -.406 | -3.211 | .002 |
|  | Depression | -.003 | .003 | -.151 | -.856 | .395 |
|  | Anxiety | .004 | .010 | .064 | .367 | .715 |
| Disgust | (Constant) | 1.241 | .280 |  | 4.430 | <.001 |
|  | GCE | -.064 | .039 | -.214 | -1.660 | .103 |
|  | Age | .026 | .011 | .286 | 2.299 | .025 |
|  | Gender | -.063 | .053 | -.161 | -1.188 | .240 |
|  | Depression | -.004 | .003 | -.235 | -1.275 | .208 |
|  | Anxiety | .001 | .010 | .025 | .140 | .890 |
| Sadness | (Constant) | 1.665 | .239 |  | 6.980 | <.001 |
|  | GCE | -.053 | .034 | -.193 | -1.539 | .129 |
|  | Age | .011 | .010 | .143 | 1.179 | .243 |
|  | Gender | -.123 | .044 | -.360 | -2.794 | .007 |
|  | Depression | -.003 | .003 | -.214 | -1.199 | .236 |
|  | Anxiety | .014 | .008 | .293 | 1.653 | .104 |
| Fear | (Constant) | 2.113 | .290 |  | 7.299 | <.001 |
|  | GCE | -.045 | .043 | -.148 | -1.037 | .304 |
|  | Age | -.011 | .012 | -.126 | -.916 | .364 |
|  | Gender | -.091 | .054 | -.250 | -1.699 | .095 |
|  | Depression | -.002 | .003 | -.133 | -.684 | .497 |
|  | Anxiety | .002 | .010 | .046 | .237 | .813 |
| Surprise | (Constant) | 1.699 | .266 |  | 6.384 | <.001 |
|  | GCE | -.059 | .037 | -.202 | -1.596 | .116 |
|  | Age | .006 | .011 | .072 | .592 | .556 |
|  | Gender | -.145 | .049 | -.382 | -2.934 | .005 |
|  | Depression | .003 | .003 | .161 | .887 | .379 |
|  | Anxiety | -.005 | .009 | -.095 | -.530 | .598 |
| Pleasure | (Constant) | 1.587 | .329 |  | 4.828 | <.001 |
|  | GCE | -.113 | .046 | -.314 | -2.471 | .016 |
|  | Age | .005 | .013 | .045 | .369 | .713 |
|  | Gender | -.146 | .061 | -.313 | -2.396 | .020 |
|  | Depression | -.003 | .004 | -.132 | -.725 | .471 |
|  | Anxiety | .002 | .011 | .036 | .203 | .840 |

*Note*. GCE, Gaze-cuing effect; SOA, stimulus onset asynchrony.

**Supplementary Table 6.** Regression results of the accuracy scores and the RTs (sensitivity) of emotion recognition as outcome variables and the GCE at the SOA of 250ms or 750ms when the gaze cue in the previous trial was congruent with the target position as a predictor, and age, gender, depression, and anxiety, as covariates.

| **SOA** | **Independent**  **Variables** | **B** | ***SE*** | ***β*** | ***t*** | ***P*** |
| --- | --- | --- | --- | --- | --- | --- |
| **Accuracy as an outcome variable** | | | | | | |
| 250ms | (Constant) | 2.802 | 1.127 |  | 2.485 | .016 |
|  | GCE | .135 | .155 | .111 | .868 | .389 |
|  | Age | -.004 | .046 | -.012 | -.092 | .927 |
|  | Gender | .149 | .204 | .096 | .727 | .470 |
|  | Depression | -.003 | .013 | -.050 | -.265 | .792 |
|  | Anxiety | -.045 | .038 | -.218 | -1.170 | .247 |
| 750ms | (Constant) | 2.772 | 1.132 |  | 2.449 | .017 |
|  | GCE | .096 | .155 | .078 | .619 | .538 |
|  | Age | -.002 | .046 | -.006 | -.044 | .965 |
|  | Gender | .151 | .206 | .098 | .733 | .466 |
|  | Depression | -.005 | .012 | -.081 | -.434 | .666 |
|  | Anxiety | -.040 | .038 | -.196 | -1.055 | .296 |
| **Sensitivity as an outcome variable** | | | | | | |
| 250ms | (Constant) | 1.575 | .230 |  | 6.855 | <.001 |
|  | GCE | -.038 | .032 | -.150 | -1.215 | .229 |
|  | Age | .007 | .009 | .096 | .779 | .439 |
|  | Gender | -.104 | .042 | -.321 | -2.508 | .015 |
|  | Depression | -.001 | .003 | -.041 | -.221 | .826 |
|  | Anxiety | <.001 | .008 | .010 | .057 | .955 |
| 750ms | (Constant) | 1.591 | .228 |  | 6.976 | <.001 |
|  | GCE | -.050 | .031 | -.191 | -1.586 | .118 |
|  | Age | .007 | .009 | .090 | .737 | .464 |
|  | Gender | -.109 | .041 | -.334 | -2.617 | .011 |
|  | Depression | -3.639 | .002 | -.003 | -.015 | .988 |
|  | Anxiety | -.001 | .008 | -.022 | -.122 | .903 |

*Note*. GCE, Gaze-cuing effect; SOA, stimulus onset asynchrony.

**Supplementary Table 7.** Regression results of the conformity effect as an outcome variable and the GCE at the SOA of 500ms when the gaze cue in the previous trial was congruent with the target position as a predictor, and age, gender, depression, and anxiety as covariates.

| **Dependent**  **Variable** | **Independent**  **Variables** | **B** | ***SE*** | ***β*** | ***t*** | ***P*** |
| --- | --- | --- | --- | --- | --- | --- |
| Social conformity | (Constant) | .011 | .260 |  | .042 | .967 |
|  | GCE | .080 | .037 | .266 | 2.160 | .035 |
|  | Age | .015 | .010 | .169 | 1.429 | .158 |
|  | Gender | -.091 | .050 | -.231 | -1.809 | .075 |
|  | Depression | <.001 | .003 | .024 | .137 | .892 |
|  | Anxiety | -.001 | .009 | -.012 | -.070 | .944 |

*Note*. GCE, Gaze-cuing effect; SOA, stimulus onset asynchrony.

**Supplementary Table 8.** Regression results of the conformity effect as an outcome variable and the GCE at the SOA of 250ms or 750ms when the gaze cue in the previous trial was congruent with the target position as a predictor, and age, gender, depression, and anxiety as covariates.

| **SOA** | **Independent**  **Variables** | **B** | ***SE*** | ***β*** | ***t*** | ***P*** |
| --- | --- | --- | --- | --- | --- | --- |
| **Social conformity as an outcome variable** | | | | | | |
| 250ms | (Constant) | .071 | .269 |  | .264 | .792 |
|  | GCE | .008 | .039 | .024 | .198 | .843 |
|  | Age | .015 | .011 | .170 | 1.376 | .174 |
|  | Gender | -.118 | .051 | -.299 | -2.330 | .023 |
|  | Depression | -.001 | .003 | -.055 | -.300 | .765 |
|  | Anxiety | .002 | .009 | .043 | .240 | .811 |
| 750ms | (Constant) | .060 | .268 |  | .224 | .824 |
|  | GCE | .025 | .038 | .080 | .669 | .506 |
|  | Age | .015 | .011 | .171 | 1.395 | .168 |
|  | Gender | -.114 | .051 | -.290 | -2.247 | .028 |
|  | Depression | -.001 | .003 | -.060 | -.335 | .739 |
|  | Anxiety | .003 | .009 | .048 | .272 | .787 |

*Note*. GCE, Gaze-cuing effect; SOA, stimulus onset asynchrony.

**Discussion**

In the present study, we hypothesized that individuals’ gaze-cuing effect (GCE) would be associated with emotion recognition ability in general and had no specific predictions about different emotions. Supporting our hypothesis, our data showed that the GCE significantly predicted the overall accuracy and sensitivity scores (i.e., averaged *d*’ value and RT across the six different emotions). However, our exploratory analyses on separate emotions revealed unexpected differences across the six emotions, which deserves further discussion. Due to the limit of space, we discuss these additional findings here. It should be noted that despite the differences in the statistical significance, the directions of effects (i.e., positive relationships between the GCE and the performance) were consistent across the six emotions.

For the accuracy score, individuals with greater GCE showed more accurate identification of the target’s sadness, fear, surprise, and pleasure but not anger and disgust. Sad and pleasant facial expressions are commonly thought to serve an emotion-sharing function in social interaction contexts (Blair, 2003), although these two emotions differ in their valence information. More specifically, sad facial expressions frequently convey information signaling losses such as losing resources, status, or close ones to others (Reed & DeScioli, 2017), and this process is likely to evoke empathic responses. Also, the expressions of happiness are suggested to signal possible positive affiliative opportunities and foster shared positive experiences with the perceiver (Garcia & Tully, 2020). Given that joint attention is a very basic form of cognitive processes necessary for experience sharing, the practice of joint attention during early development could provide a foundation for the ability to share sad or pleasant affects and efficiently process the affective information, resulting in more accurate recognition of such expressions.

Fearful and surprised facial expressions are both characterized by wide-eyed expressions (Kim et al., 2004; Whalen et al., 2004) and recognizing those emotions heavily relies on processing information from the eye region (Adolphs et al., 2005; Li et al., 2018). For example, previous studies have reported a larger amount of time fixating on the eyes when fearful expression was displayed (Scheller et al., 2012) and fewer errors in recognizing surprise when looking longer at the target’s eye region (Bal et al., 2010). We used gaze-cuing paradigm to assess individuals’ GCE, in which each individual’s attention was influenced by others’ gaze. Considering above, the use of this paradigm might have had advantages in identifying how the sensitivity to the target’s gaze interacts with emotional expressions conveyed primarily by eyes including fear and surprise. More detailed information related to visual processing using eye tracking may help clarify the nature of their relationships.

Although the direction of effects was consistent across different emotions, the positive relationship between the GCE and the accuracy of emotion recognition was not significant in anger and disgust. One of the possible reasons for such results may be a ceiling effect. For example, angry faces, due to their threat-related value, are known to attract automatic attention and enhance performance in various tasks (Belopolsky et al., 2011; Feldmann‐Wüstefeld et al., 2011; Pedale et al., 2017). The same might be the case for disgusted face, given its evolutionary value (Curtis et al., 2011; Tybur et al., 2013). Higher performance consistent across individuals may result in a ceiling effect, yielding insufficient individual variabilities in the outcome variable (i.e., accuracy score) to produce significant correlations with the GCE score. To explore this possibility, we examined the hit rate and accuracy score for each of the six emotional faces (Table S2). Indeed, the average hit rate and accuracy score for angry faces was higher than other emotional faces except for happy faces. However, the average hit rate and accuracy score were the highest for happy faces among the six emotions followed by angry faces, and the standard deviations of the accuracy scores for angry faces and for happy faces were comparable to each other. Furthermore, both the hit rate and accuracy score for disgusted faces were not high enough to be considered for a ceiling effect and the standard deviation was comparable to that of other emotions. Additionally, the scatter plots of the GCE index and the accuracy scores for angry and disgusted faces did not show any patterns indicating a ceiling effect or particularly low individual variabilities.

For the sensitivity (i.e., RT), we found that the GCE predicted greater sensitivity (i.e., shorter RT) in recognizing the target’s emotions including pleasure and anger. That is, individuals with greater GCE were faster in detecting the target’s angry and pleasant expressions even when the intensity was relatively subtle or moderate. One possible explanation could be found in the research on face in the crowd effect (Becker et al., 2011; Hansen & Hansen, 1988; Pinkham et al., 2010), which suggests that happy and angry faces are detected more rapidly in the social context. Researchers have suggested that the rapid detection of both threatening (i.e., angry face) and non-threatening (i.e., happy face) expressions of social partners is important for survival. Also, happy and angry faces are associated with approach and avoidance motivations (Nikittin & Freund, 2010). Therefore, it would be plausible that individuals with greater GCE, who tend to pay more attention to social cues in general, are more attuned to the facial emotions conveying such important information for further social interactions.

Given that each emotional expression has its unique developmental trajectory (Rodger et al., 2015), our finding indicates the possibility that the dynamic practice of joint attention at early developmental stages might contribute to the accurate and sensitive recognition of specific emotional expressions. However, this assumption should be treated with caution because prior work has shown that autistic individuals who are frequently characterized with difficulties in joint attention were consistently slower in recognizing all types of emotions than typically developing children (Bal et al., 2010; Georgopoulos et al., 2022). Additionally, most relevant research has used static face presentation rather than a face with gradually increasing intensity of expression. Therefore, additional research is needed to address how commonly and differently joint attention influences recognition of different emotions.

**References**

Adolphs, R., Gosselin, F., Buchanan, T. W., Tranel, D., Schyns, P., and Damasio, A. R. (2005). A mechanism for impaired fear recognition after amygdala damage. *Nature* 433, 68−72.

Bal, E., Harden, E., Lamb, D., Van Hecke, A. V., Denver, J. W., and Porges, S. W. (2010). Emotion recognition in children with autism spectrum disorders: relations to eye gaze and autonomic state. *J. Autism Dev. Disord.* 40, 358−370.

Becker, D. V., Anderson, U. S., Mortensen, C. R., Neufeld, S. L., and Neel, R. (2011). The face in the crowd effect unconfounded: happy faces, not angry faces, are more efficiently detected in single- and multiple-target visual search tasks. J. Exp. Psychol. Gen. 140, 637–659.

Belopolsky, A. V., Devue, C., and Theeuwes, J. (2011). Angry faces hold the eyes. *Vis. Cogn.* 19, 27–36.

Blair, R. J. R. (2003). Facial expressions, their communicatory functions and neuro–cognitive substrates. *Philos. Trans. R. Soc. Lond., B, Biol. Sci.* 358, 561−572.

Curtis V, de Barra M, Aunger R. (2011). Disgust as an adaptive system for disease avoidance behaviour. *Philos. Trans. R. Soc. Lond. B. Biol. Sci.* 366, 389–401.

Feldmann‐Wüstefeld, T., Schmidt‐Daffy, M., and Schubö, A. (2011). Neural evidence for the threat detection advantage: differential attention allocation to angry and happy faces. *Psychophysiology* 48, 697–707.

Garcia, S. E., and Tully, E. C. (2020). Children’s recognition of happy, sad, and angry facial expressions across emotive intensities. *J. Exp. Child Psychol.* 197, 104881.

Georgopoulos, M. A., Brewer, N., Lucas, C. A., and Young, R. L. (2022). Speed and accuracy of emotion recognition in autistic adults: the role of stimulus type, response format, and emotion. *Autism Res.* 15, 1686−1697.

Hansen, C. H., and Hansen, R. D. (1988). Finding the face in the crowd: an anger superiority effect. *J. Pers. Soc. Psychol.* 54, 917–924.

Kim, H., Somerville, L. H., Johnstone, T., Polis, S., Alexander, A. L., Shin, L. M., and Whalen, P. J. (2004). Contextual modulation of amygdala responsivity to surprised faces. *J. Cogn. Neurosci.* 16, 1730−1745.

Li, S., Li, P., Wang, W., Zhu, X., and Luo, W. (2018). The effect of emotionally valenced eye region images on visuocortical processing of surprised faces. *Psychophysiology* 55, e13039.

Nikittin, J., and Freund, A. M. (2010). A motivational perspective on reactions to emotional faces. In A. Freitas-Magalhães (Ed.), *Emotional expression: The brain and the face* (pp. 85–108). Edições Universidade Fernando Pessoa.

Pedale, T., Basso, D., and Santangelo, V. (2017). Processing of negative stimuli facilitates event-based prospective memory only under low memory load. *J. Cogn. Psychol.* 29, 920–928.

Pinkham, A. E., Griffin, M., Baron, R., Sasson, N. J., and Gur, R. C. (2010). The face in the crowd effect: anger superiority when using real faces and multiple identities. *Emotion* 10, 141–146.

Reed, L. I., and DeScioli, P. (2017). The communicative function of sad facial expressions. *Evol. Psychol.* 15, 1474704917700418.

Rodger, H., Vizioli, L., Ouyang, X., and Caldara, R. (2015). Mapping the development of facial expression recognition. *Dev. Sci.* 18, 926–939.

Scheller, E., Büchel, C., and Gamer, M. (2012). Diagnostic features of emotional expressions are processed preferentially. *PLoS One* 7, e41792.

Tybur, J. M., Lieberman, D., Kurzban, R., & DeScioli, P. (2013). Disgust: evolved function and structure. *Psychol. Rev.* 120, 65–84.

Whalen, P. J., Kagan, J., Cook, R. G., Davis, F. C., Kim, H., Polis, S., McLaren, D. G., Somerville, L. H., McLean, A. A., Maxwell, J. S. and Johnstone, T. (2004). Human amygdala responsivity to masked fearful eye whites. *Science* 306, 2061−2061.
